# Morphological differences between coastal bottlenose dolphin (*Tursiops aduncus*) populations identified using non-invasive stereo-laser photogrammetry

**DOI:** 10.1038/s41598-019-48419-3

**Published:** 2019-08-22

**Authors:** Martin van Aswegen, Fredrik Christiansen, John Symons, Janet Mann, Krista Nicholson, Kate Sprogis, Lars Bejder

**Affiliations:** 1 Environmental and Conservation Sciences, Murdoch University, South Street, Murdoch, WA 6150 Australia; 2 Aquatic Megafauna Research Unit, Harry Butler Institute, Murdoch University, South Street, Murdoch, WA 6150 Australia; 3 Marine Mammal Research Program, Hawaii Institute of Marine Biology, University of Hawaii at Manoa, 96744 Kaneohe, USA; 4 Aarhus Institute of Advanced Studies, Høegh-Guldbergs Gade 6B, 8000 Aarhus, Denmark; 5 Zoophysiology, Department of Bioscience, Aarhus University, 8000 Aarhus, Denmark; 6 Department of Biology and Department of Psychology, Georgetown University, 20057 Washington, DC USA

**Keywords:** Behavioural ecology, Ecophysiology, Animal physiology, Marine biology, Evolutionary ecology

## Abstract

Obtaining morphometric data on free-ranging marine megafauna is difficult, as traditional methods rely on post-mortem or live-capture techniques. We linked stereo-laser photogrammetry with long-term demographic data to compare length-at-age (LaA) growth curves of two well-studied populations of Indo-Pacific bottlenose dolphins (*Tursiops aduncus*) in south-western (SW) and Shark Bay (SB), mid-western Australia. First, we determined the relationship between total length (TL) and blowhole-to-dorsal fin (BH-DF) length from post-mortem subjects (R^2^ = 0.99, n = 12). We then predicted TL from laser-derived BH-DF measurements of 129 and 74 known-age individuals in SW and SB, respectively. Richards growth models best described our LaA data. While birth length (103–110 cm) was similar between study regions, TL estimates at 1, 3, 12, and 25 years differed significantly (p < 0.001). Asymptotic length of adult males (SW = 246 cm, SB = 201 cm) and females (SW = 244 cm, SB = 200 cm) also differed significantly. Morphotypic variations likely reflect regional adaptations to local water temperatures, with the temperate SW having cooler waters than sub-tropical SB. We demonstrate the effectiveness of a non-invasive technique to understand ecological, demographic and life-history characteristics of long-lived marine megafauna, which are critical parameters for informing conservation and management actions.

## Introduction

A comprehensive understanding of population-specific demographics, life-history traits and behavioural ecology is essential for the effective management of long-lived, slow-reproducing species^1,2^. Such parameters are linked to morphological and age-specific processes, often regulated by physiological, ecological, evolutionary, and anthropogenic factors^3^. Morphometric data of cetaceans contribute to the assessment of individual- and population-based reproduction^4,5^, health^6,7^ and demography^8–10^ and in conjunction with genetic information, taxonomic status^11,12^. Despite its many applications, obtaining accurate morphometric data on free-ranging cetaceans is challenging.

Traditionally, three approaches have been used to obtain morphometric data on cetaceans: post-mortem specimens^13^, live captive study subjects^14,15^, and capture-release programs^16,17^. Post-mortem specimens are usually sourced from stranding events^18^ in addition to incidental^19^ and deliberate kills^20^. The dependence on post-mortem study subjects has the disadvantage of reliance on unpredictable access to animals and small sample sizes^21^. In addition, post-mortem specimens may provide a biased sample, if animals of specific age, sex, size or health are more likely to strand, be incidentally caught or killed^22^. Capture-release programs provide a unique opportunity to repeatedly measure individuals over time but come with both considerable ethical and logistical considerations^17^.

In recent decades, photogrammetry has emerged as an alternative morphometric technique, highlighted by its application in studies of animal populations in both terrestrial^23–25^ and marine environments^26–28^. Stereo-photogrammetry, where two parallel cameras capture a composite image simultaneously, is considered one of the earliest forms of photogrammetry. However, its practicality is limited by the cumbersome nature of the required hardware^29,30^. Stereo-laser photogrammetry is a popular alternative to stereo-photogrammetry, due to its simplicity. The technique consists of two perfectly parallel laser dots calibrated at a specific distance apart (e.g. 10 cm), thereby providing a known-length scale within a photograph which allows the size of animals in an image to be measured (i.e. by converting measured pixels to centimetres)^8^. The technique also allows for photo-identification data to be obtained simultaneously, so that a specific measurement can be linked to a particular individual^31,32^.

We used stereo-laser photogrammetry to obtain total length (TL) estimates of coastal Indo-Pacific bottlenose dolphins (*Tursiops aduncus*) from south-western (SW) and Shark Bay (SB), Western Australia. Laser-derived measurements, in conjunction with available long-term demographic records of individual dolphins, were applied to develop length-at-age (LaA) growth curves for each region (SW and SB). Growth parameter estimates derived from growth models were used to characterise and compare growth adaptations, with the aim of quantifying potential differences in the morphology of *T. aduncus* from two geographically separated regions.

## Results

### Relationship between blowhole-to-dorsal fin length and total length

Physical measurements of blowhole-to-dorsal fin length (BH-DF) and total length (TL) were obtained on 12 post-mortem individuals (males n = 6, females n = 6) stranded in SW Australia. The significant positive relationship between BH-DF and TL (in centimetres) was then used to estimate the TL of *T. aduncus* in both SW and SB regions (F_1,10_ = 1341, p < 0.001, R^2^ = 0.992, TL = 5.0583 + 3.17 × BH-DF, Supplementary Fig. S1). While our sample size was small (n = 12), there is evidence to suggest the relationship between BH-DF and TL can be generalized across both *T. aduncus* and *Tursiops truncatus*^33^ (Supplementary Fig. S1).

### Description of length-at-age data obtained in south-west and Shark Bay

For the SW region, laser-derived measurements were collected during 40 boat-based surveys in Bunbury (n = 28) and Mandurah (n = 12). Of the 2,103 photographs taken, 828 photographs were of sufficient quality for analyses. A total of 129 individual dolphins were identified and measured, including 56 females, 39 males, and 34 of unknown-sex. An average of 6.4 measurements were available for each individual (SE = 0.37), with a mean coefficient of variance (CV) of 1.9% estimated for repeated TL estimates of the same individuals across multiple photographs (range = 0.02–6.68%). Minimum age estimates for females ranged from three days old to 29 years (Supplementary Fig. S2a), with laser-derived TL estimates ranging between 106.1 cm and 256.8 cm (Supplementary Fig. S3a, Table S1). Minimum age estimates for males ranged from four days old to 29 years (Supplementary Fig. S2a), with a TL range of 105.7–254.4 cm (Supplementary Fig. S3a, Table S1). No significant differences in TL were observed between males (n = 10, mean = 243.7 cm, SE = 3.1 cm) and females (n = 13, mean = 242.9 cm, SE = 2.1 cm) over the age of 20 (p = 0.832).

In SB, stereo-laser photogrammetry data were collected during boat-based surveys (n = 11 days) and beach food-provisioning events (n = 10 days). Over this period, 732 photographs were taken, with 355 images (boat = 216, beach = 139) of sufficient quality to warrant inclusion for further analyses. Of the 74 individuals sampled, 42 were female, 24 were male, and 8 were of unknown sex, with an average of 4.8 measurements per individual (SE = 0.81). A mean CV of 1.7% was estimated for repeated TL estimates of the same individuals across multiple photographs, with a range of 0.03–8.8%. Minimum age estimates for females ranged from 1.7 to 44 years (Supplementary Fig. S2b), with TL estimates ranging from 139.9 to 210.5 cm (Supplementary Fig. S3b, Table S1). Minimum age estimates for males ranged between 3.8 and 41 years (Supplementary Fig. S2b), with a TL range of 157.8–209.9 cm (Supplementary Fig. S3b, Table S1). The youngest SB individual was < 2 weeks old, with a TL estimate of 102.8 cm (Table S1), and of unknown sex. Like the SW region, no sexual dimorphism was detected in SB individuals >20 years old (p = 0.084), with males averaging 202.9 cm (n = 11, SE = 1.25 cm) and females 198.7 cm (n = 13, SE = 1.92 cm).

### Selection of the best-fitting growth models

Of the four growth models fitted to the LaA data, Richards growth model (RGM) best described *T. aduncus* growth in both study regions. For the SW sample, the RGM provided the best fit (w_*i*_ = 1.00, Supplementary Table S2). Visual inspection of the Typical von Bertalanffy (TvB), Original von Bertalanffy (OvB) and Gompertz function (GOM) growth curves highlighted the lack of model fit for younger individuals in the SW sample (Supplementary Fig. S4a), with the flexible RGM curve best fitting the observed data at younger ages. For the SB sample, the RGM received moderate support (w_*i*_ = 0.49), suggesting the OvB (w_*i*_ = 0.22) and TvB (w_*i*_ = 0.22) models also fitted the data reasonably well (Supplementary Table S2). Despite stronger overlapping of candidate growth curves observed for *T. aduncus* in the SB region (Supplementary Fig. S4b), only the most parsimonious model (RGM) was used to infer estimates of growth.

### Estimation of biological parameters

**Figure 1:**
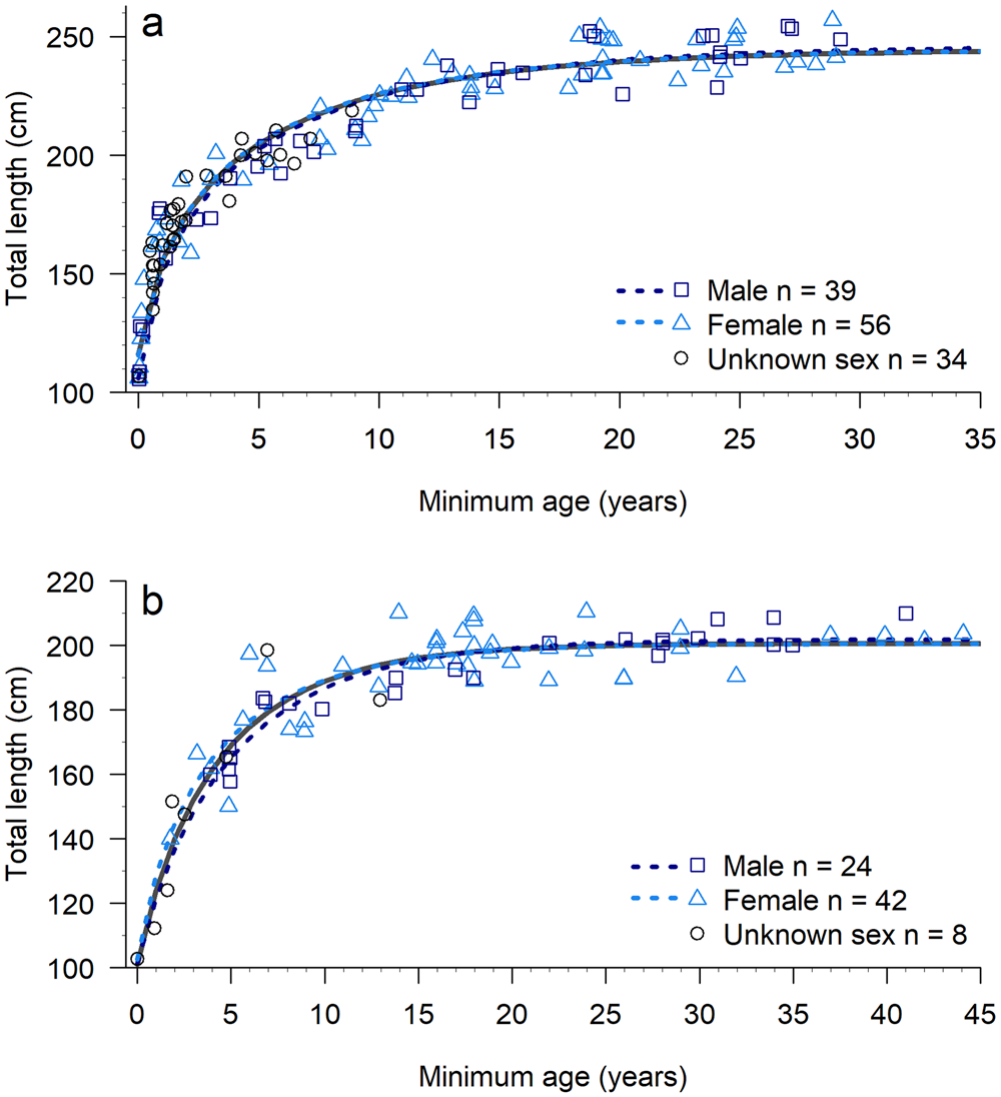
Richards growth curves for bottlenose dolphins (*Tursiops aduncus*) sampled in (**a**) south-western Australia (n = 129) and (**b**) Shark Bay (n = 74). Growth curves for both-sexes combined (solid), males (dashed dark blue) and females (dashed light blue) are shown, with male (dark blue square), female (light blue triangle) and unknown-sex (open circles) dolphins fitted using one randomly selected measurement per individual.

All SW calves less than two-weeks old measured between 106.1–110.7 cm (mean = 107.6 cm, SD = 1.87 cm, n = 6). Both SW and SB growth curves were characterised by rapid early growth before slowing as juveniles approached maturity (Fig. 1a,b). Southwest calves exhibited a distinct period of accelerated growth during their first year (Fig. 1a), with the RGM predicting a median TL of 155.9 cm (95% CI = 155.3–156.5 cm) at age 1 year. By the end of their third year, SW calves were estimated to have a median TL of 187.1 cm (95% CI = 186.5–187.7 cm). Median TL values of 120.1 cm (95% CI = 118.4–122.2 cm) and 149.5 cm (95% CI = 148.2–151.1 cm) were predicted for SB calves aged 1 and 3 years, respectively.

The RGM predicted a slightly larger L_∞_ estimate for SW males (246.1 cm, 95% CI = 239.2–254.5 cm, Fig. 1a, Supplementary Table S1) relative to SW females (244.5 cm, 95% CI = 239.9–250.7 cm). Shark Bay males reached an asymptote at 201.9 cm (95% CI = 199.2–205.1 cm, Fig. 1b, Supplementary Table S1) and for SB females, the RGM produced a median L_∞_ estimate of 200.5 cm (95% CI = 196.7–205.3 cm, Supplementary Table S1).

### Estimating age and length at independence and first reproduction

**Figure 2:**
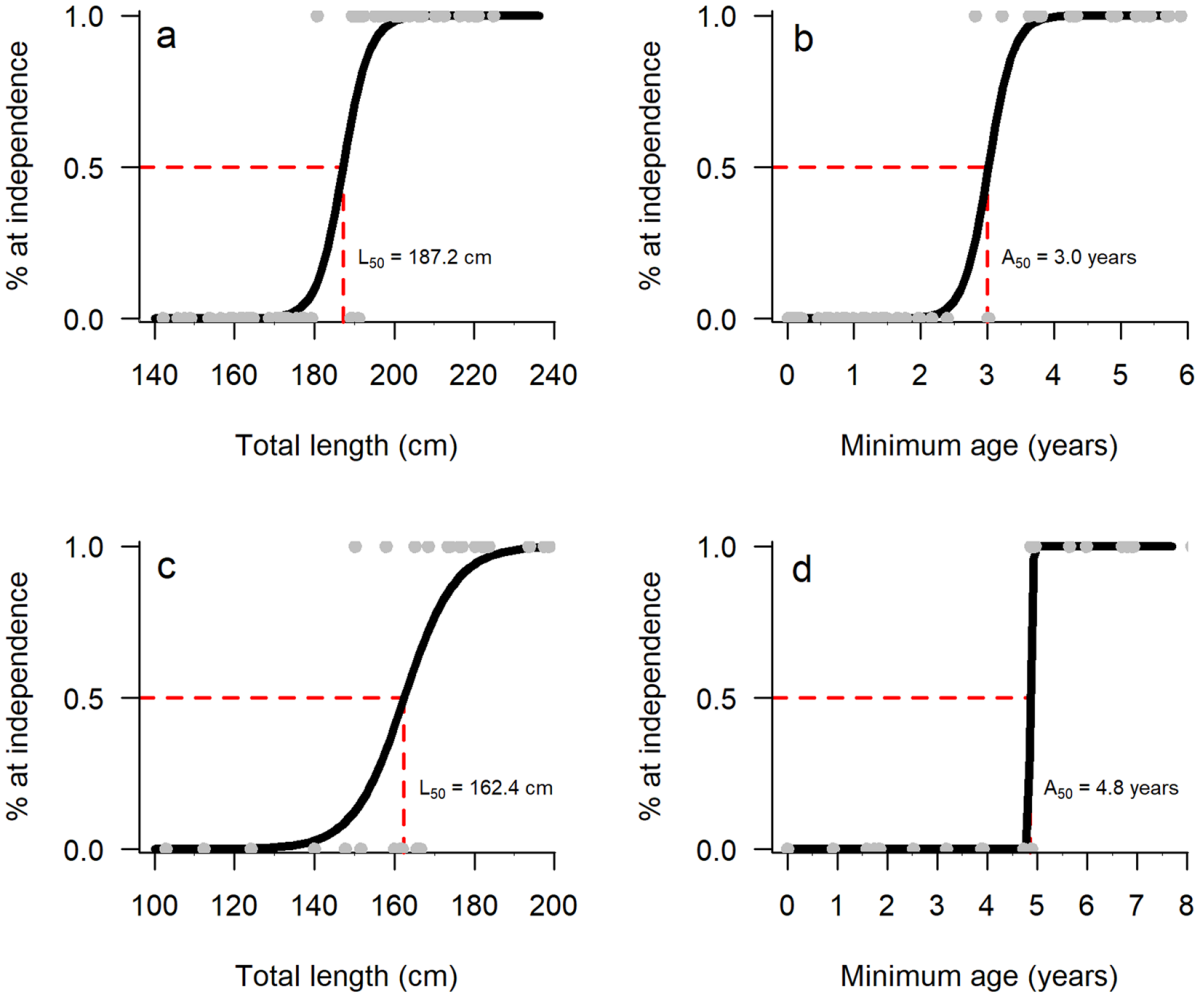
Logistic curves displaying the mean total length (L_50_) and age (A_50_) estimates at which 50% of individuals are predicted to be independent. Plots ‘a’ and ‘b’ show respective L_50_ and A_50_ estimates for the southwest region (n = 77), with plots ‘c’ and ‘d’ representing Shark Bay (n = 27). The grey points represent individuals sampled.

For SW and SB, age and length had a significant effect on the probability of a dolphin becoming independent and reproducing for the first time (p < 0.001, Supplementary Table S3). In the SW sample containing 45 dependent calves and 32 independent juveniles, 24 were female, 19 male and 34 of unknown sex. Fifty percent of SW calves were estimated to become independent by 3.0 years (95% CI = 2.5–3.3 years, Fig. 2b). The SB sample consisted of 11 dependent calves and 16 independent juveniles, including 11 females, nine males and seven dolphins of unknown sex. The A_50_ estimate for SB calves was 4.8 years (95% CI = 4.3–4.9 years, Fig. 2d). Despite becoming independent earlier, SW calves exhibited a larger L_50_ value (187.2 cm, 95% CI = 180.0–191.1 cm, Fig. 2a) relative to SB dolphins (162.4 cm, 95% CI = 151.9–168.2 cm, Fig. 2c).

**Figure 3:**
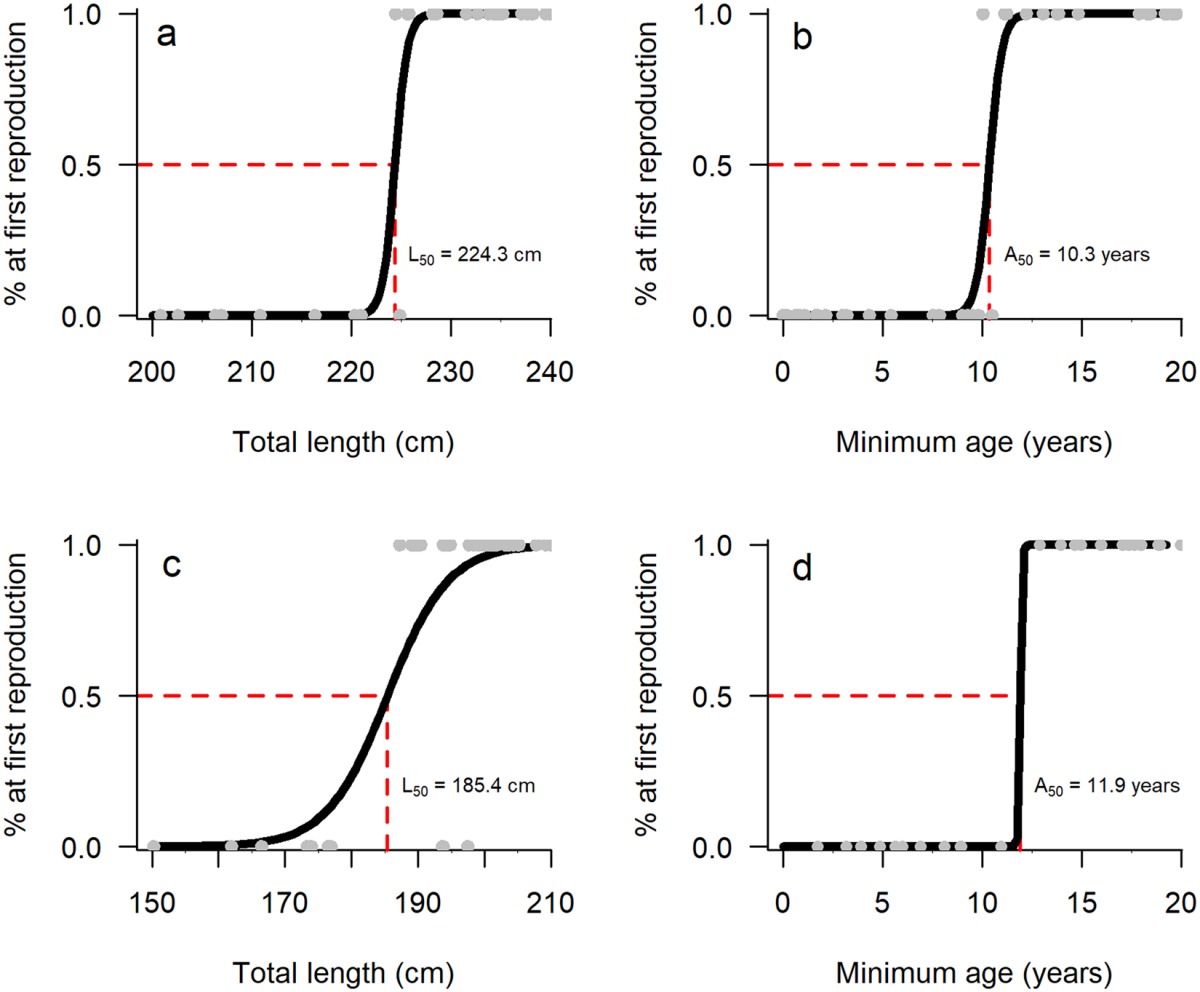
Logistic curves displaying the mean total length (L_50_) and age (A_50_) estimates at which 50% of females are predicted to reproduce for the first time. Plots ‘a’ and ‘b’ show respective L_50_ and A_50_ estimates for southwest females (n = 56), with plots ‘c’ and ‘d’ representing their Shark Bay conspecifics (n = 42). The grey circles represent individual females sampled.

For first reproduction, the SW sample consisted of 32 females with previous calving histories and 24 females never observed with a calf. In SB, 31 females had previous calving histories while 11 females exhibited no evidence of previous birth at the time of measurement. Southwest females attained A_50_ at a younger age (10.3 years, 95% CI = 9.6–11.3 years, Fig. 3b) than their SB conspecifics (11.9 years, 95% CI = 10.8–12.9 years, Fig. 3d). First reproduction L_50_ estimates calculated for SW and SB were 224.3 cm (95% CI = 220.9–226.4, Fig. 3a) and 185.4 cm (95% CI = 181.3–190.0 cm, Fig. 3c), respectively.

### Sensitivity analysis: accounting for measurement and age-estimation errors

We investigated the influence of error on our LaA estimates using maximum error values for both laser-derived measurements and individual age estimations. When stereo-laser images were obtained at 15° from perpendicular, a dolphin replica experiment yielded a mean measurement error of 1.27% (SD = 0.69, Supplementary Fig. S5) equating to 0.84 cm (range = 0.29–1.47 cm). At this angle, precision was high between length measurements from 45 non-sequential images (CV = 1.31%).

Age-estimation error distributions were greatest for SW individuals over the age of 20, with far greater age certainty for calves and juveniles (Supplementary Fig. S6a). Age estimations of SB dolphins were of better quality with no individuals assigned a maximum age of 45, reflected by reduced horizontal error distributions (Supplementary Fig. S6b). The profiles of both SW and SB RGM curves remained relatively unchanged following the resampling procedure, with little deviation from either of the original RGM curves (Supplementary Fig. S6). Each of the estimated LaA values displayed narrow distributions (Supplementary Fig. S7) and HPD intervals (Supplementary Table S4), indicating both SW and SB RGM models were robust to potential error present in this study.

### Regional differences in dolphin morphology

**Figure 4:**
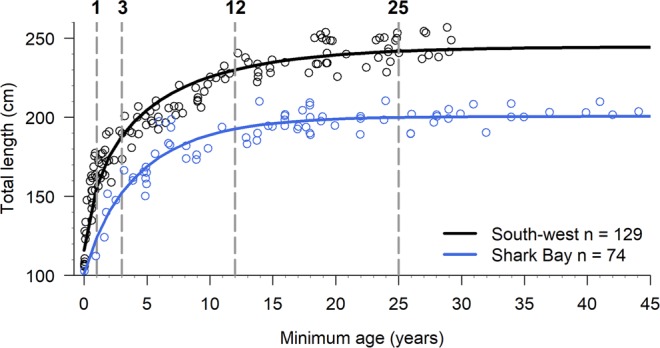
Comparison of regional growth curves. Overlaid Richards growth curves demonstrating differences in length-at-age of bottlenose dolphins (*Tursiops aduncus*) between south-western Australia (black; males, females and unknown sexes) and Shark Bay (blue; males, females and unknown sexes). Points represent individual dolphins and dashed grey vertical lines indicate the four age-classes that were compared: 1, 3, 12 and 25 years. Observe the distinct difference in first-year growth between the two study regions.

Bootstrapped TL estimates of dolphins differed significantly between the two study regions, with SB dolphins being significantly shorter across ages 1, 3, 12 and 25 years (p < 0.001 for all four tested ages, Fig. 4). Total-length differences across the four age classes ranged from 35.8 cm (age 1 year) to 39.2 cm (age 25 years), with a median of 36.8 cm (SD = 1.58 cm, Supplementary Table S4). This variation in age-specific growth can be visualised by the lack of overlapping distributions in each age class (Supplementary Fig. S8).

## Discussion

In this study we measured body morphometrics using non-invasive laser photogrammetry, to characterise and compare the growth of *T. aduncus* from two well-studied populations in Western Australia. We identified marked differences in the growth between the SW and SB, with *T. aduncus* in the SW being significantly longer in body length than their SB conspecifics. This difference in length was not caused by regional variation in birth size, but by a distinct difference in growth across all life stages (i.e. neonates, calves, juveniles and adults).

Latitudinal differences in body size have been well documented in *Tursiops* spp., with larger body sizes typically reported in cooler regions^14,34–36^. A recent study by Cheney *et al*.^33^ demonstrated the potential of laser photogrammetry by developing the first laser-derived LaA growth curves for a bottlenose dolphin population in a temperate environment. Using these measurements, the study reported adult *T. truncatus* inhabiting temperate waters were approximately 30% longer than estimates produced for adult *T. truncatus* in sub-tropical waters^17^. Using the same approach, we detected regional differences in body length across all life stages, with SW *T.aduncus* being up to 20% longer than SB dolphins. By identifying morphological differences between two populations of *T. aduncus*, we add further support for the accuracy and applicability of laser photogrammetry as a morphometric tool.

Coastal sea surface temperatures (SSTs) off Bunbury (in the SW) range between 14 °C (June to August, austral winter) and 23 °C (December-March, summer), with an annual mean SST of 18 °C^37,38^. In Monkey Mia, SSTs range between 16 °C (June to August, winter) and 30 °C (December-March, summer), with a mean annual SST of 22 °C^39^. While minimum SSTs are similar between study sites, long-term data^39^ (2009–2019) indicate the mean winter SST for Monkey Mia (19 °C) is greater than the mean annual SST reported for Bunbury (18 °C)^37,38^.

The inverse relationship between body size and surface water temperature is characteristic of Bergmann’s rule^40^, which describes a trade-off between surface area and volume. The surface area of an endothermic animal represents its ability to dissipate heat, while its volume serves as a measure of its heat generation capability^41^. A reduced surface-area-to-volume ratio is thus considered a selective advantage, enabling large-bodied animals residing in cooler environments to regulate body heat more efficiently^42,43^. While SST is likely the most dominant factor affecting body size between *T. aduncus* in SB and SW, our findings likely reflect a complex interplay of phenotypic and genotypic factors, which can only be elucidated using a combination of environmental, morphometric and genetic approaches^11^.

Total length estimates of all seven SW and SB neonate calves less than two-weeks old (2 females, 3 males, 2 unknown-sex) ranged between 102.8 cm and 110.7 cm (mean = 106.9 cm). These values complement birth length estimates of *T. aduncus* in Tanzania (103 cm)^44^, and *T. truncatus* in South Africa (103–111 cm)^45–47^ and the Gulf of Mexico (103–109 cm)^48^. Remote estimates of newborn length are rare, with Cheney *et al*.^33^ reporting laser-derived estimates for *T. truncatus* in Scotland of 128–188 cm for calves in their first three months. Length estimates of neonatal *T. aduncus* are typically limited to post-mortem observations with lengths between 93–121 cm recorded for South Australian *T. aduncus* younger than three months (n = 10, mean = 104.5 cm)^49^. While our TL estimates represent the first laser-derived length measurements of neonatal *T. aduncus*, repeat measurements of young calves over time will provide a more comprehensive profile of neonatal growth. Non-invasive morphometric techniques also reduce dependence on deceased specimens, which may not be representative of wild populations.

Using the mean birth lengths derived from this study, SW neonates (107.6 cm) were estimated to increase in length by 48.3 cm (44.8%) by the end of their first year, while SB calves (102.8 cm) only increased by 17.3 cm (16.8%). Although regional differences in asymptotic length are evident, caution is recommended when interpreting the lack of accelerated growth observed in SB calves. Additional sampling of SB calves would be beneficial in confirming whether such large differences in first year growth are robust, or due to limited calves sampled in SB (n = 11) relative to SW (n = 45).

Our age-at-independence estimates for both SW (3.0 years) and SB (4.8 years) fall within the range previously reported for *T. aduncus* (2.5–8.5 years)^50–53^. Our A_50_ estimate is slightly larger than the mean age at independence previously reported for SB dolphins (3.98 years)^50,53^, and is more reflective of the age at independence for last-born SB calves^53^ (4.86 years). Our study is the first to report laser-derived L_50_ estimates for *T. aduncus*, with SW dolphins becoming independent at a larger body length than SB dolphins (L_50_: SW = 187.2 cm and SB = 162.4 cm). The ability to quantify individual and population-level calf growth promotes the value of stereo-laser photogrammetry, with the potential to investigate the influence of maternal investment on calf size and associated fitness consequences in future studies.

Age at first reproduction estimates for female *T. aduncus* vary considerably across studies (7–15 years)^14,44,45,50,54^, with our SW A_50_ estimate (10.3 years) within the range of those previously reported. In SB, 42% of first births occur by age 12^53,55^, validating our SB A_50_ estimate of 11.9 years. Like independence, our estimates of age and length at first reproduction indicate SW females to be younger, but larger the first time they give birth (SW = 10.3 years and 224.3 cm, SB = 11.9 years and 185.4 cm). Our length-at-first reproduction estimates are the first to be derived from live, free-ranging dolphins using non-invasive laser photogrammetry. These estimates are similar to those reported for captive and post-mortem *Tursiops* spp. females from temperate (227–238 cm)^14,34^ and tropical waters globally (190–200 cm)^44,54^. In our study, SW mothers may be maximising the reproductive fitness of their offspring by providing the maternal investment necessary to attain optimal independence and first reproduction sizes at earlier ages, given faster growing mammals tend to mature earlier than slower-growing mammals^18,56,57^.

Stereo-laser photogrammetry shows promise in its ability to detect individual variations in growth, providing opportunities to elucidate the effects of maternal investment and experience on calf growth and survival. Cheney *et al*.^33^ recently demonstrated the efficacy of this technique by investigating the fitness implications of variable calf length, reporting first-born calves were shorter than calves of experienced mothers and, more importantly, that calf length was a significant predictor of first-year mortality. These results suggest calf growth may be a valuable proxy of maternal investment and condition, with future studies recommended to incorporate laser photogrammetry into long-term monitoring efforts^33,58^.

In both SW and SB, L_∞_ estimates were slightly larger for males than females. Despite this, no significant differences in TL were detected in either SW or SB adults over the age of 20. While adult male *T. aduncus* are typically heavier than adult females^45^, little to no sexual dimorphism has been detected using TL^14,35,45^. Since we could not estimate mass using stereo-laser photogrammetry, we were unable to investigate regional growth characteristics and sexual dimorphism using a combination of TL and mass.

Our L_∞_ estimates for *T. aduncus* SW males (246.1 cm) and females (244.5 cm) are slightly larger than those reported for male (243 cm) and female (238 cm) *T. aduncus* in South Africa^45^ and *T. truncatus* in near-shore waters off Perth, Western Australia (~240 cm)^14^. Interestingly, our L_∞_ estimates for SB male (201.9 cm) and female (200.5 cm) *T. aduncus* are among the shortest reported for this species. Adults of similar size have been documented from deceased subjects in the Arafura Sea, Northern Australia (214 cm, 11°42′S, 137°14′E^34^, Spencer Gulf, South Australia (214 cm, 32°29′S, 133°17′E)^54^ and Zanzibar, Tanzania (222 cm, 06° S, 39° E)^44^. While a few qualitative accounts have briefly described the small size (~200 cm) of adult SB dolphins^50,59,60^, this study is the first to measure and estimate the size and growth of coastal dolphins in both SW and SB.

The mean measurement error in the dolphin replica experiment (1.27% at 15° horizontal angle) was within the error range previously reported for stereo-laser photogrammetry (1.2–3.5%)^27,28,32,33^. Precision was also high, with mean CV estimates achieved in both the error experiment (1.3%) and field-based laser photogrammetry (1.7–1.9%) comparing favourably to previous laser photogrammetry (0.7–3.7%)^28,31,32^, stereo-photogrammetry (4.3%)^29^ and aerial photogrammetry studies (<2%)^61,62^.

Our sensitivity analysis demonstrated all four LaA estimates (i.e. ages 1, 3, 12, 25 years) were robust to measurement and age estimation errors. Age estimations for all SB individuals were accurate to within three years. Age estimation for SW individuals under 12 years of age were also accurate (median = 2 months, range = 1 day to 4 years). The narrow density distributions produced by the sensitivity analysis showed the SW RGM model remained robust with LaA values easily within the typical length variation reported in mature *Tursiops* spp.^17^ (Supplementary Fig. S8). This is because all SW individuals first sighted as adults were positioned at asymptotic regions, as opposed to earlier regions, of the RGM. This suggests any influence on the RGM output would have been minimal, as *T. aduncus* growth usually ceases by the age of 15^45,50^. This approach, however, may not be appropriate for immature individuals who are still subject to growth. Nonetheless, results from our sensitivity analysis propose that it may be possible to investigate individual and population-specific growth using incomplete demographic data on older individuals. The sensitivity analysis also confirms the high accuracy of our stereo-laser photogrammetry approach in obtaining accurate body morphometric measurements of *T. aduncus* and consequent growth curves.

Repeated measurements of known-age individuals over time will yield more comprehensive information on the variability of individual and population-level growth rates, and establish an ideal platform for the investigation of genetic, biological, ecological and anthropogenic factors influencing growth. Our ability to quantify differences in growth over a relatively small geographical distance demonstrates the value of using this technique to investigate body size at various ages and life history stages (birth, independence, first reproduction and physical maturity). Stereo-laser photogrammetry, therefore, provides a valuable opportunity to collect morphometric data on free-ranging cetacean populations in an accurate, non-invasive manner, which can ultimately inform conservation management strategies.

## Methods

### Study locations and dolphin populations

**Figure 5:**
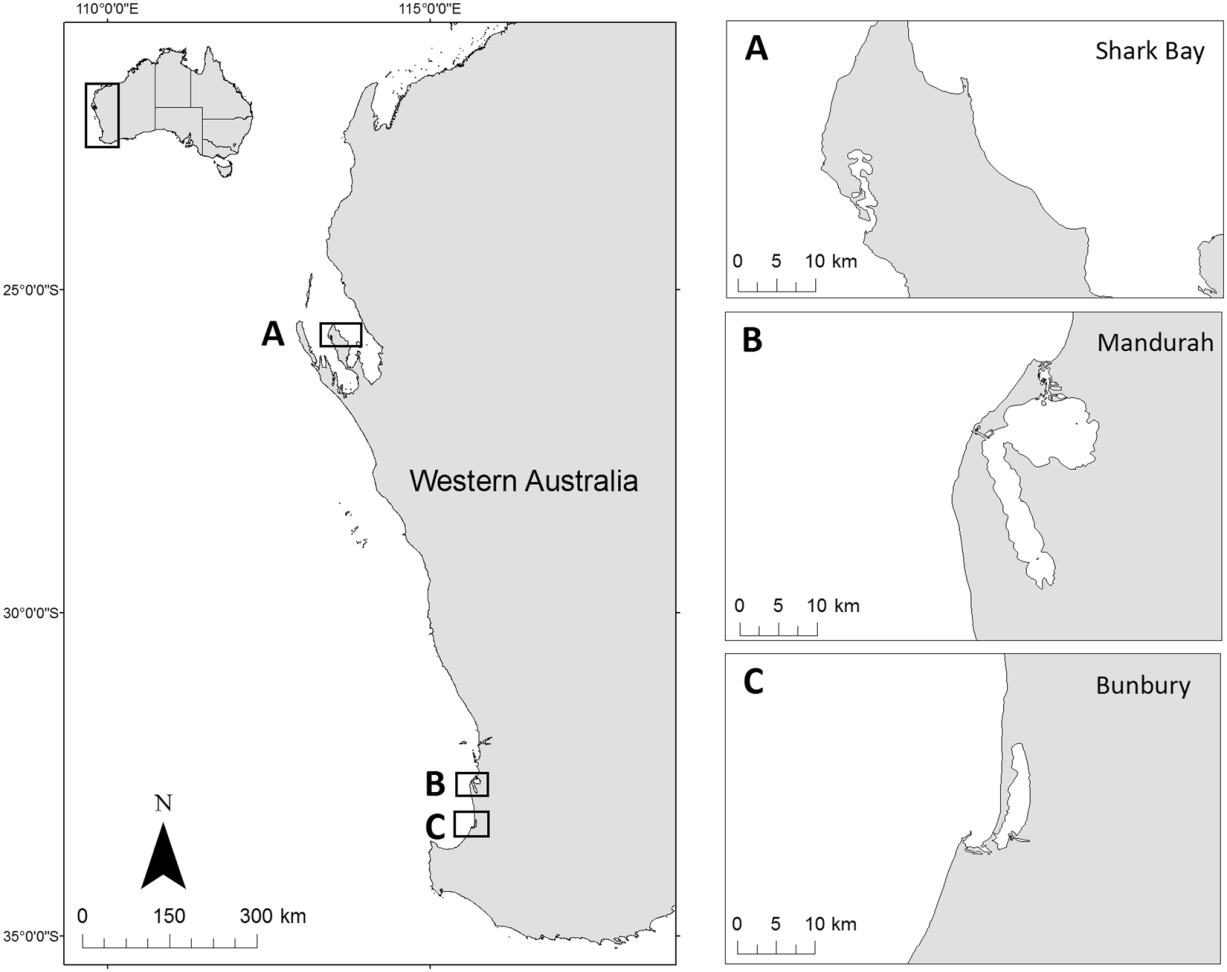
Map of study regions. The study regions in Western Australia encompassed (**A**) Shark Bay (SB), and the south-west (SW) comprised of two study locations (**B**) Mandurah and (**C**) Bunbury. The straight-line distance between Shark Bay and Bunbury is 860 km.

Between May 2016 and March 2017, three study sites along the Western Australian coast were used to collect stereo-laser photogrammetry data on *T. aduncus* (Fig. 5). The SW region comprised of dolphins sampled in both Bunbury (33°32′S, 115°63′E, Fig. 5C) and Mandurah (32°32′S, 115°44′E, Fig. 5B). The distance between these locations is approximately 95 km. Both locations exhibit an identical Mediterranean climate with temperate coastal environments^38,63^. The SB region comprised of dolphins sampled off Monkey Mia (25°47′S, 113°43′E, Fig. 5A), located in the Eastern Gulf of SB, approximately 860 km north-west of Bunbury. This study was approved by the Western Australian Department of Biodiversity, Conservation and Attractions (SF010738, CE005422), with all fieldwork conducted in accordance to standards set by the Murdoch University Ethics Committee (R2649/14).

### Age estimates for south-west and Shark Bay individuals

#### Bunbury

Since 2007, dedicated year-round photo-identification surveys have been conducted off Bunbury as part of the South West Marine Research Program (SWMRP). The SWMRP has identified approximately 500 individual dolphins, facilitating the development of a long-term demographic dataset comprising of photo-identification^64^, demographic (age and sex)^65^ and sighting-history information (Supplementary Table S5). Additional photo-identification, demographic and sighting data have been obtained on food-provisioned dolphins since 1989, following the establishment of a not-for-profit dolphin-tourism centre conducting dolphin-provisioning and eco-tours^66^.

#### Mandurah

The Mandurah Dolphin Research Project (MDRP) commenced in 2016, with approximately 500 dolphins subsequently identified (Supplementary Table S5). Additional long-term demographic are available through collaborative citizen science and historic live-stranding records dating back to 1987 (Supplementary Table S5).

For SW (Bunbury and Mandurah) calves (0–3.0 years) and juveniles (3.5–10 years), minimum and maximum age estimates were established based on long-term sighting records of both the individual dolphin in question and its mother. For example, the minimum age of an individual was derived from the date it was first sighted, with its maximum age calculated using the date the mother was last sighted with either a sibling calf or no calf present. For some SW individuals first sighted as adults, minimum age was calculated from the date the dolphin was first sighted, with a maximum age of 45 assigned corresponding to the accepted life expectancy of *T. aduncus*^45^. While minimum age may underestimate the true age of an individual first sighted as an adult, physical maturity (cessation of growth due to fusing of vertebral epiphyses) usually occurs between 10–15 years^45,67,68^. This ultimately supports the notion that growth in length would be minimal (or non-existent) for individuals first identified as adults.

#### Shark Bay

Dolphin food-provisioning has occurred in Monkey Mia since 1964, with some demographic data recorded prior to the commencement of governmental monitoring in the 1980s (Supplementary Table S5). The Shark Bay Dolphin Project (SBDP) has identified over 1,600 individual dolphins since 1984^69^, making it the second-longest-running dolphin research program worldwide^70^. The estimated birth date of each SB dolphin was assigned to one of four accuracy categories, including day, week, month and year estimates. These categories represent age estimates accurate to within seven days, four weeks, one year and three years, respectively. For each SB dolphin sampled, minimum and maximum age estimates were calculated using the accuracy category assigned to that individual.

#### Stereo-laser photogrammetry system

Total length (tip of rostrum to tail notch) is a fundamental morphometric parameter in marine mammal life history studies^71^. With direct estimates of TL being difficult to acquire on free-ranging dolphins^33^, morphometric indices such as BH-DF are instead frequently employed to estimate TL, using well-established allometric relationships derived through stranding and post-mortem subjects^15,29,33^. Since the BH-DF region is regularly visible when a dolphin surfaces, the BH-DF measurement is the most practical means of estimating TL from boat-based platforms.

**Figure 6:**
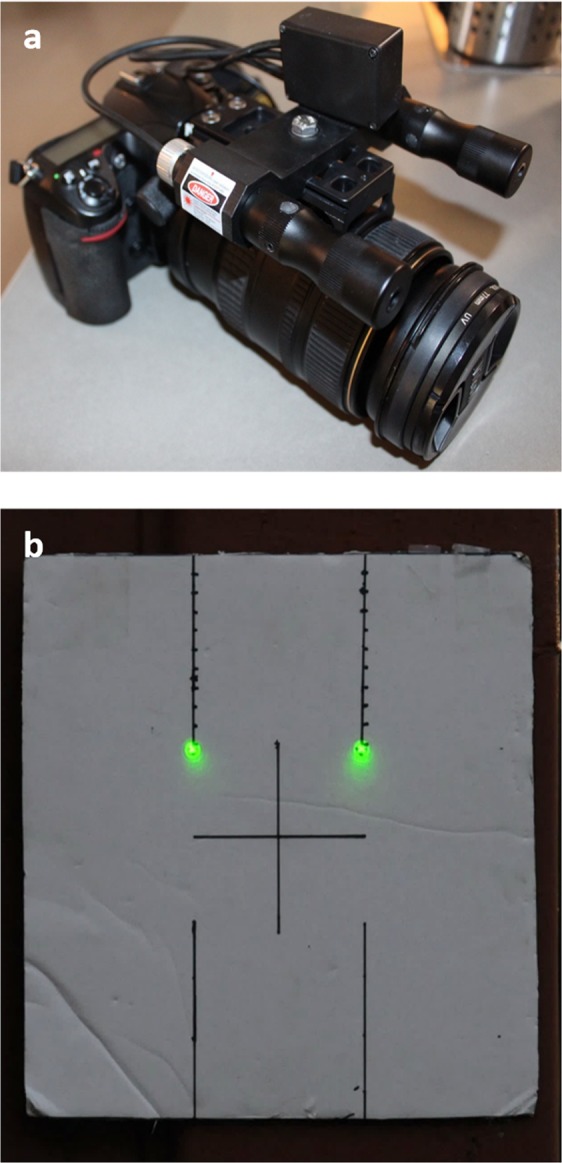
The stereo-laser photogrammetry system used in this study. (**a**) The DSLR camera with the mounted stereo-laser system; and (**b**) the calibration board used to calibrate the paired Beamshot lasers. The distance between the two green laser dots is 10 cm.

To estimate the BH-DF length of *T. aduncus*, a stereo-laser system was used in combination with a 12.3 MP Nikon D300s camera body equipped with a Nikon 80–400 mm f/4.5–5.6D ED lens. The laser system consisted of a custom-made aluminium block housing two Beamshot (Quarton USA Inc, USA; 5 mW; 532 nm) laser modules separated by a 10 cm distance and attached to the camera lens using a tripod mount (Fig. 6a)^33^. To improve safety around research personnel and dolphins, the laser system was activated and deactivated using an electronic switch box (see Cheney *et al*.^33^ for a detailed description of the laser system). The laser system was designed, manufactured and supplied by Barnacle Electronics, Scotland^33^. To ensure the lasers remained parallel at 10 cm apart, calibration photographs were taken at five incremental distances (5–25 m) before and after each boat survey. If the laser dots did not align with the reference points on the calibration board (Fig. 6b), adjustments were made by rotating the vertical and horizontal-configured grub screws on the laser modules.

#### Field methods

Laser-derived data were collected during boat-based photo-identification surveys, conducted in Bunbury and Mandurah between 2016 and 2017. In Shark Bay, laser-derived data were collected over a three-week period in October 2016, using both boat-based surveys and beach food-provisioning events. All boat-based surveys were conducted using small research vessels less than 5.5 m in length, powered by 60–100 hp outboard engines. Once a dolphin group was sighted, the research vessel was positioned parallel to the targeted group, at distances of 5–25 m. Photographs were taken of surfacing dolphins with the photographer using the camera autofocus point within the viewfinder to place the laser sights on the dorso-lateral surface of the dolphin. All sampled individuals were subsequently identified using dorsal fin photo-identification^72^ records specific to each population.

#### Image processing

**Figure 7:**
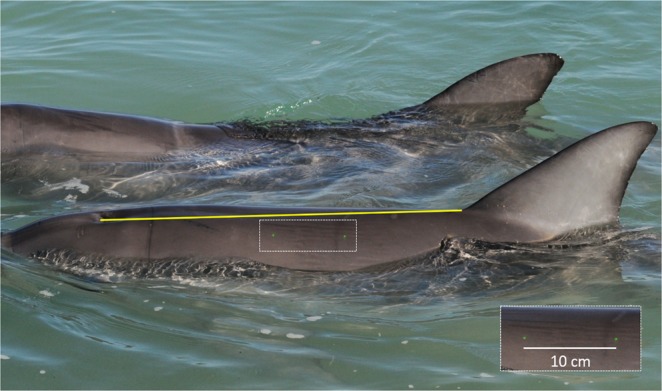
An example of a good-quality photograph for stereo-laser photogrammetry. In our study, this requires a bottlenose dolphin (*Tursiops aduncus*) positioned perpendicular to the camera, displaying both the blowhole and anterior origin of dorsal fin landmarks (the yellow line joins the two). Both laser dots are visible, with the inset image providing an enlarged view of the two laser dots positioned 10 cm apart.

To date, no means of quantifying the horizontal angle within single-camera photographs have been developed. Consequently, rigorous image selection was considered best practice in reducing horizontal angle error. To be included in this study, photographs were required to be in-focus, displaying both laser dots clearly, with the dolphin positioned as close to parallel to the camera as possible (Fig. 7; Supplementary Figs. S5 and S9). Additionally, the blowhole and dorsal fin of the dolphin needed to be unobstructed for both measurement and identification purposes.

All measurements were made using the free image processing software *ImageJ*^73^. The number of pixels between the medial point of the blowhole (BH) and the anterior origin of the dorsal fin (DF, BH-DF) were converted into centimetres, using the 10 cm scale signified by the distance between the two laser dots. The BH-DF lengths of individuals were converted into TL estimates using the linear relationship between BH-DF and TL derived from post-mortem individuals (Supplementary Fig. S1).

#### Growth curve analysis and estimation of growth parameters

To describe the growth of *T. aduncus*, four common non-linear growth functions were applied to derived LaA data. Length-at-age data for each region (SW and SB) were subset to estimate male, female and combined-sex asymptotic lengths (L_∞_) separately. The first two candidate models consisted of the Original (OvB, Eq. 1) and Typical (TvB, Eq. 2) forms of the von Bertalanffy growth function^74^. The OvB is considered more appropriate for marine mammals as length-at-birth (L_0_) can be estimated following a gestational period^75^. The final two candidate models included the Gompertz function^76^ (GOM, Eq. 3), and the Richards growth model^77^ (RGM, Eq. 4). The shape parameter (*p*) within the RGM enables the inflection point of the curve to be set anywhere between the range of minimum and maximum asymptote values, providing additional flexibility. The respective equations for these growth functions are as follows:

1 $${\rm{Original}}\,{\rm{von}}\,{\rm{Bertalanffy}}\,({\rm{OvB}}):\,{{\rm{L}}}_{t}={{\rm{L}}}_{\infty }-({{\rm{L}}}_{\infty }-{{\rm{L}}}_{0})\ast {{\rm{e}}}^{(-K\ast t)}$$

2 $${\rm{Typical}}\,{\rm{von}}\,{\rm{Bertalanffy}}\,({\rm{TvB}}):\,{{\rm{L}}}_{t}={{\rm{L}}}_{\infty }\,(1-{{\rm{e}}}^{(-K(t-{t}_{0}))})$$

3 $${\rm{Gompertz}}\,({\rm{GOM}}):\,{{\rm{L}}}_{t}={{\rm{L}}}_{\infty }\ast \,{\rm{e}}{(}^{-e({gi}(t-{t}_{0}))})$$

4 $${\rm{Richards}}\,({\rm{RGM}}):\,{{\rm{L}}}_{t}={{\rm{L}}}_{\infty }{(1-{{\rm{e}}}^{(-K(t-{t}_{0}))})}^{p}$$

where L_*t*_ denotes length-at-age *t*,

L_∞_ is the asymptotic average length,

*K* is the Brody growth rate coefficient,

*t*_0_ is an artificial modelling artefact representing time when average length is zero,

*t* is a theoretical function of time and age,

L_0_ is the mean length at time zero (birth),

*gi* is the instantaneous growth rate at the inflection point; and

*p* is an artificial modelling artefact determining the shape of the curve.

To reduce the likelihood of overparameterization within the RGM, some starting values for the non-biological parameters ‘*t*_0_’ and ‘*p*’ were fixed to constant values^78^ (Supplementary Table S6). All candidate models were fit using the ‘FSA’^79^ and ‘nlstools’^80^ packages in R version 3.2.4^81^ with median L_∞_ estimates and 95% confidence intervals obtained through bootstrap resampling (1,000 iterations). Models were compared using Akaike Information Criterion (AIC)^82^, where each model was ranked relative to the best fitting model using corrected delta AIC values (AIC_*c*_) in the ‘AICmodavg’ package^83^. Akaike model weights (w_*i*_) were used to determine which of the four growth functions provided the best fit for LaA data obtained on dolphins in the SW and SB regions.

#### Estimating age and length at independence and first reproduction

We estimated the mean length (L_50_) and age (A_50_) at which 50% of dolphins were predicted to be (a) independent, and (b) reproductive for the first time (females only). These estimates were fit separately for SW and SB regions using binomial generalized linear models (GLMs), with a logit link function. All GLMs were fit using the ‘FSA’^79^ and ‘car’^84^ packages in R. Logistic regression was used to calculate the respective L_50_ and A_50_ estimates, by applying Eq. (5) ^85,86^:

5 $${{\rm{A}}}_{\mathrm{50}}\,{\rm{and}}\,{{\rm{L}}}_{{\rm{50}}}=\frac{\mathrm{log}(\frac{p}{1-p})-{\rm{\alpha }}}{{{\rm{\beta }}}_{1}}$$

where *p* is the probability of ‘success’, 1 − *p* is the probability of ‘failure’, and; α and β are fitting constants^85^. To calculate L_50_ and A_50_ at independence, ‘success’ and ‘failure’ were defined as being ‘independent’ or ‘dependent’, respectively. For L_50_ and A_50_ at first reproduction, ‘success’ and ‘failure’ were defined as being ‘mature’ or ‘immature’, respectively. To quantify the uncertainty around the estimates, 95% confidence intervals were produced through bootstrap resampling (1,000 iterations).

Independence: Every individual with a laser-derived TL estimate and minimum age was assigned a binary value based on their dependence (‘0’) or independence (‘1’) status at the time of sampling. The status of each individual was determined using long-term demographic and sighting information specific to each population, including repeated sightings of calves in infant position, mother-calf association patterns and birth records of the mother. For example, individuals were assigned a value of ‘0’ if they were consistently sighted in infant position or sighted in close association with their mother (i.e. no repeated observations of separation between mother and calf). Individuals assigned a value of ‘1’ displayed marked changes in mother-calf association patterns, were sighted >5 times on their own or with other juveniles or their mother was repeatedly observed with a new sibling calf.

First reproduction: Combined samples of reproductively immature and mature female individuals were used to estimate the mean length and age at which 50% of females were predicted to reproduce for the first time. Using long-term demographic and sighting records, females with confirmed calving histories (i.e. repeated sighting of the female with a dependent calf) were assigned a value of ‘1’ and individuals never observed in close association with a calf were given a value of ‘0’.

#### Sensitivity analysis: accounting for measurement and age-estimation errors

We investigated the extent to which errors influenced the predicted LaA values. Errors were attributed to two factors: errors in laser-derived length measurements and uncertainty in age estimation of individual dolphins. Multiple laser photogrammetry studies have highlighted the potential influence that the horizontal angle between the photographer and the subject (i.e. dolphin) could have on measurement accuracy^8,27,31^. Thus, to examine the extent to which horizontal angle influenced laser-derived length estimates, we conducted a measurement error experiment using a three-dimensional dolphin replica model (*T. aduncus* courtesy of the Western Australian Department of Biodiversity, Conservation and Attractions). The model was rotated in 15° horizontal angle increments, ranging from perpendicular (hereafter referred to as 0°) to 75° from perpendicular to the camera (see Supplementary Fig. S9a). Eighteen photographs (with the laser dots positioned on the dolphin model) were taken at each angle increment, enabling the known BH-DF length of the replica model to be measured using nine non-sequential images. This process was repeated utilising five-metre distance increments between 5 and 25 m (Supplementary Fig. S9b). The laser-derived length measurement error estimate was calculated as the mean percentage error obtained at 15° from perpendicular (the greatest horizontal angle permissible in image selection). To assess the influence of age estimation error, we used the minimum and maximum age values assigned to each individual sampled in the study.

Bootstrap resampling was used to calculate median TL at ages 1, 3, 12, and 25 years, by resampling the laser-derived measurement (in cm) and age estimate (in years) of each individual by 1,000 iterations, and then re-fitting the chosen growth model. These simulated values were visually displayed in a square-shaped error distribution around each true data point, with the height and width representing maximum measurement and age-estimation error for each dolphin, respectively. From the output density distributions, 95% highest posterior density (HPD) intervals were calculated.

#### Regional differences in dolphin morphology

Intra-specific differences in total length were investigated using median LaA estimates obtained from the sensitivity analysis procedure. Using years 1, 3, 12, and 25, we compared dolphin length across periods of both rapid growth and growth approaching an asymptote, while also accounting for regions of the curve where physical maturity has been reached. Years 3 and 12 also coincide with major life history events for *T. aduncus*: independence (~3–4 years)^50^ and the attainment of sexual maturity (~10–12 years)^45,50,71^, respectively. The HPD distributions obtained from the sensitivity analyses were plotted against each other, as the degree of overlapping between the LaA distributions would indicate whether significant differences were present in each age class. Welch two sample t-tests were carried out on each age class (α = 0.05), using the median LaA estimates as the dependent variable.

## Supplementary information

Supplementary Info

## Data Availability

The datasets generated during and/or analysed during the current study are available from the corresponding author on reasonable request.
