# Supplementary material for: Morphological differences between coastal bottlenose dolphin (Tursiops aduncus) populations identified using non-invasive stereo-laser photogrammetry
Source: Sci Rep. 2019 Aug 22;9:12235. doi: 10.1038/s41598-019-48419-3 (PMC6706378; doi:10.1038/s41598-019-48419-3)
Supplement: Supplementary file 1 — Supplementary Info [file 41598_2019_48419_MOESM1_ESM.pdf]

Scientific Reports

Morphological differences between coastal bottlenose dolphin (*Tursiops aduncus*) populations identified using non-invasive stereo-laser photogrammetry

Martin van Aswegen, Fredrik Christiansen, John Symons, Janet Mann, Krista Nicholson, Kate Sprogis & Lars Bejder

Corresponding author: M van Aswegen (Email: mvanas@hawaii.edu)

Electronic supplementary material:

Table. S1. Summary of minimum, maximum and asymptotic length estimates

Table. S2. Summary of corrected Akaike's information criterion model selection output

Table. S3. Length and age at 50% independence and first reproduction

Table. S4. Length-at-age estimates and HPD values from the sensitivity analysis

Table. S5. Summary of study sites, research programs and individuals identified

Table. S6. Summary of RGM parameter estimates - L_{∞}, K, t_0 and p

Fig. S1. Relationship between total length and blowhole-to-dorsal fin length

Fig. S2. Age-frequency distributions of dolphins sampled in SW and SB study sites

Fig. S3. Length-frequency distributions of dolphins sampled in SW and SB study sites

Fig. S4. Visual representations of each of the candidate model fits for SW and SB sites

Fig. S5. Measurement error results from the 3D dolphin replica dolphin experiment

Fig. S6. Sensitivity analysis: measurement and age-estimation error distributions

Fig. S7. Sensitivity analysis: frequency distributions of model parameter estimates

Fig. S8. Density distributions of length-at-age values across ages: 1, 3, 12, and 25 years

Fig. S9. Example of the 3D replica dolphin experiment used to test angle and distance

Supplementary Table S1. Summary of minimum (L_{min}) and maximum (L_{max}) total lengths estimated for male (M), female (F) and unknown-sex (U) Indo-Pacific bottlenose dolphins (*Tursiops aduncus*) from south-west and Shark Bay regions. Asymptotic length (L_{∞}) estimates predicted by the Richards growth model (RGM) are also shown with 95 % confidence intervals. Both south-west and Shark Bay RGMs were subset to investigate male and female growth, as well as for all sampled individuals combined (A).

Region	Sex	n	L_{min} (cm)	L_{max} (cm)	L_{∞} (cm)	L_{∞} 95 % CI (cm)
South-west	M	39	105.7	254.4	246.1	239.2 – 254.5
	F	56	106.1	256.8	244.5	239.9 – 250.7
	U	34	106.9	218.7	-	-
	A	129	-	-	244.5	240.6 – 248.6
Shark Bay	M	24	157.8	209.9	201.9	199.2 – 205.1
	F	42	139.9	210.5	200.5	196.7 – 205.3
	U	8	102.8	198.6	-	-
	A	74	-	-	200.6	198.1 – 203.4

Supplementary Table S2. Summary of corrected Akaike's information criterion (AIC_c) model selection output for the four candidate growth models: The Richards (RGM), original von Bertalanffy (OvB), typical von Bertalanffy (TvB) and Gompertz (GOM), used to describe the length-at-age data obtained from the south-west and Shark Bay regions. The most parsimonious models are highlighted in bold.

Region	Candidate model	AIC_c	$\Delta\ AIC_c$	w_i	Log Likelihood
South-west	**RGM**	**961.13**	**0.00**	**1**	**-477.47**
	TvB	1003.74	42.61	0	-497.71
	OvB	1003.74	42.61	0	-497.71
	GOM	1013.00	51.86	0	-502.34
Shark Bay	**RGM**	**522.98**	**0.00**	**0.49**	**-257.20**
	OvB	524.64	1.65	0.22	-258.03
	TvB	524.64	1.65	0.22	-258.03
	GOM	526.81	3.82	0.07	-259.11

Supplementary Table S3. Mean total length (L_{50}) and age (A_{50}) estimates at which 50% of individuals are predicted to be independent and reproductive for the first time. Logistic regression models were run for both south-west (SW) and Shark Bay (SB) regions. Both adult male and female dolphins (A) were used in independence analyses, with only female (F) dolphins used to estimate first reproduction values. A significance level of 0.05 was used to determine the effect of age and total length on the probability of a dolphin being independent or reproductive for the first time. *** $p < 0.001$.

Parameter	Region	Sex	n	A_{50} (95% CI) (years)	Sig.	L_{50} (95% CI) (cm)	Sig.
Independence	SW	A	77	3.01 (2.58-3.39)	***	187.20 (180.07-191.18)	***
	SB		27	4.86 (4.37-4.92)	***	162.45 (151.97-168.27)	***
First reproduction	SW	F	56	10.35 (9.65-11.33)	***	224.38 (220.94-226.45)	***
	SB		42	11.91 (10.51-12.92)	***	185.40 (181.39-190.08)	***

Supplementary Table S4. Summary of median length-at-age estimates and HPD values derived from the sensitivity analysis. Age-specific regional differences in total length and their Welch Two-Sample t-test significance levels are included ($p = 0.05$). Total length at age x is represented as TLx, for example, total length at age 1 = TL_1. *** $p < 0.001$.

Region	TL_1 (cm) (95 % HPD)	TL_3 (cm) (95 % HPD)	TL_{12} (cm) (95 % HPD)	TL_{25} (cm) (95 % HPD)
South-west	155.9 (155.3-156.5)	187.1 (186.5-187.7)	228.0 (227.6-228.4)	239.3 (238.8-239.9)
Shark Bay	120.1 (118.4-122.2)	149.5 (148.2-151.1)	192.0 (191.5-192.6)	200.1 (199.5-200.6)
Δ TL (cm)	35.8	37.6	36.0	39.2
Significance	***	***	***	***

Supplementary Table S5. Study sites and the estimated number of individual dolphins identified, in addition to the commencement years and *ad-hoc* data collection. Periods of lasermetric data collection for the current study are also included.

Site	Commencement of dedicated dolphin research (year)	*Ad-hoc* data available from (year)	Number of individuals identified	Laser-derived data collection period
Bunbury	2007	1989	500[1]	May 2016 – January 2017
Mandurah	2016	1987	500[2]	June 2016 – March 2017
Shark Bay	1984	1960s	1,600[3]	October 2016

[1] J. Symons (August, 2016, pers. comm)

[2] K. Nicholson (March, 2018, pers. comm)

[3] Karniski et al. (2015)

Supplementary Table S6. Mean parameter estimates for asymptotic length (L_∞) and Brody growth coefficient (*K*) derived from Richards growth models (RGM) fitted to length-at-age data for male (M) and female (F) *Tursiops aduncus* in south-west and Shark Bay study regions. Richards growth model parameter estimates for all sexes combined (male, female and unknown-sex individuals) are also included. Parameters t_0 and *p* are modelling artefacts with no biological meaning, with some starting values fixed (*) to shape the growth curve.

Region	Sex	n	L_∞ (cm)	*K* (yr^{-1})	t_0	*p*
South-west	M	39	246.1	0.11	-0.30*	0.24*
	F	56	244.5	0.13	-0.37*	0.24*
	A	129	244.5	0.12	-0.38*	0.24*
Shark Bay	M	24	201.9	0.17	-1.90*	0.53*
	F	42	200.5	0.17	-0.88	0.35
	A	74	200.6	0.19	-1.50	0.50*

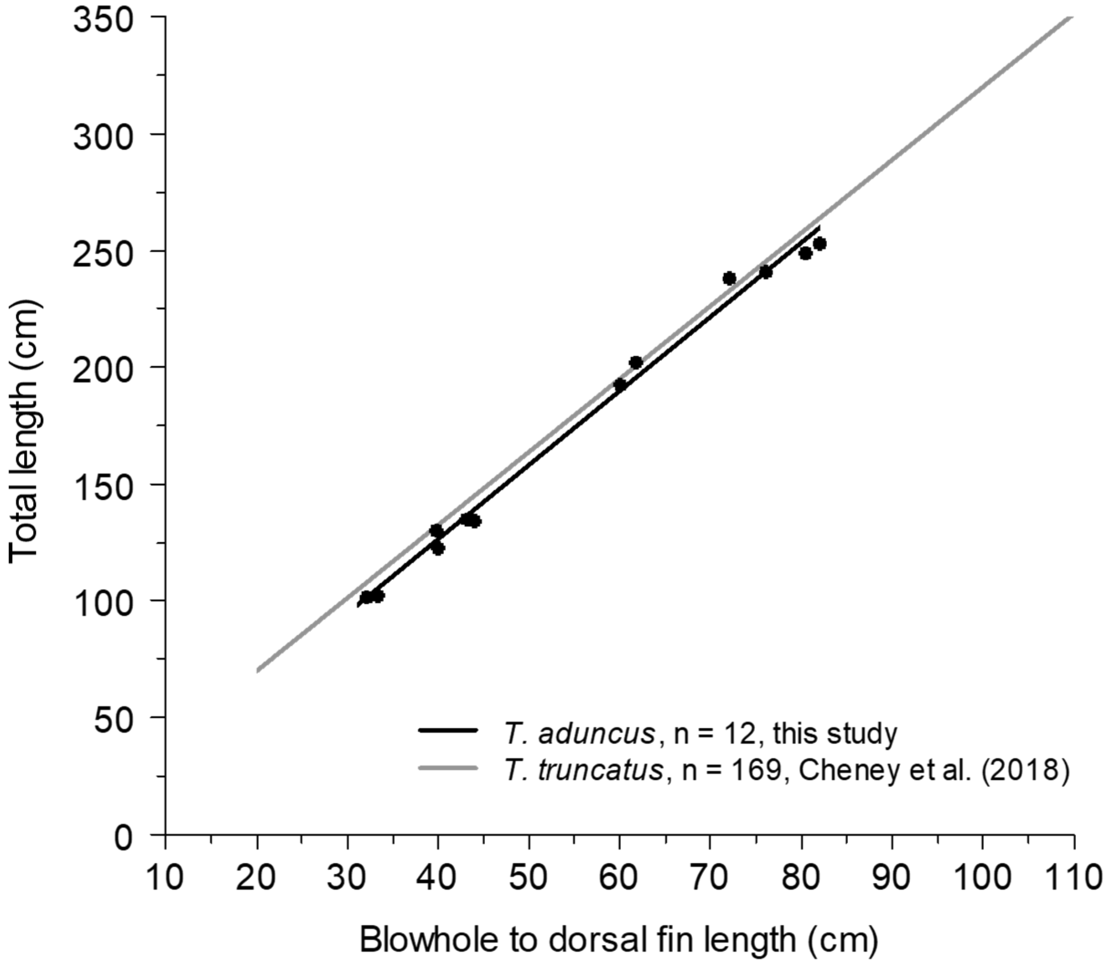

Supplementary Fig. S1. Linear relationship between total length (TL) and blowhole-to-dorsal fin (BH-DF) length for stranded *Tursiops aduncus* (n = 12, black points and line) in south-western Australia ($F_{1,10} = 1341$, $P < 0.001$, $R^2 = 0.992$, TL = 5.0583 + 3.17 × BH-DF). The grey line depicts the BH-DF to TL relationship for *Tursiops truncatus* (n = 169, $R^2 = 0.964$, y = 3.1314x+7.0626) presented in Cheney et al. 2018.

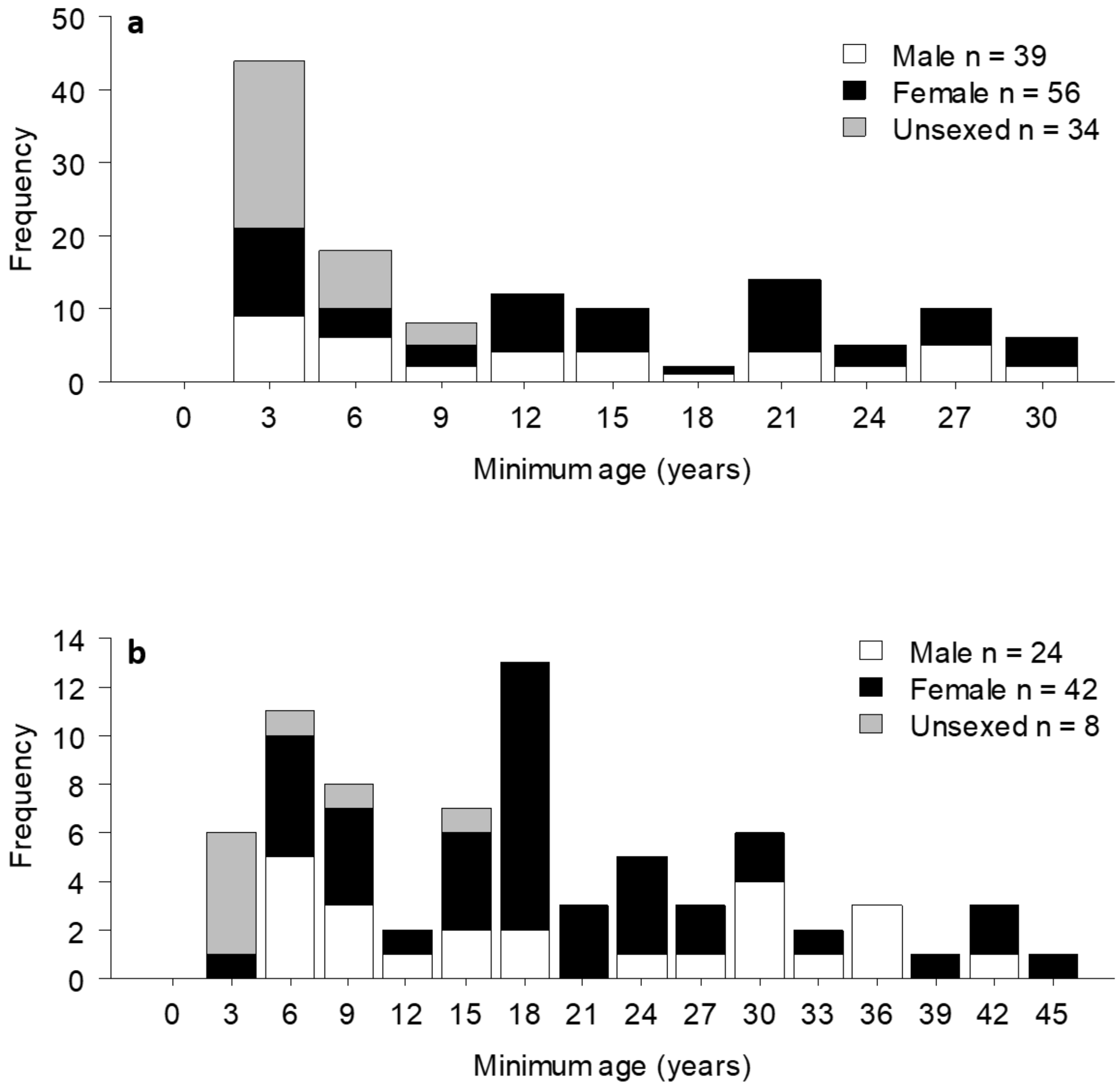

Supplementary Fig. S2. Age-frequency distributions of male (white), female (black) and unknown-sex (grey) bottlenose dolphins sampled in a) south-west and b) Shark Bay study sites. Age bins represent the three years prior to the bin in question, e.g. the '3' bin represents ages from 0 to 3 years.

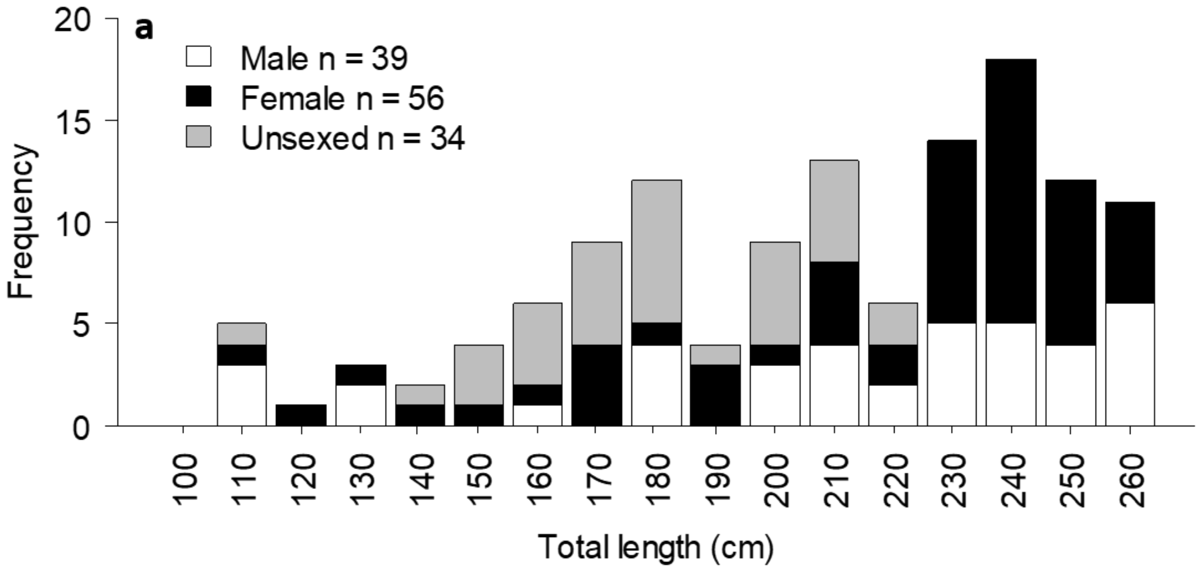

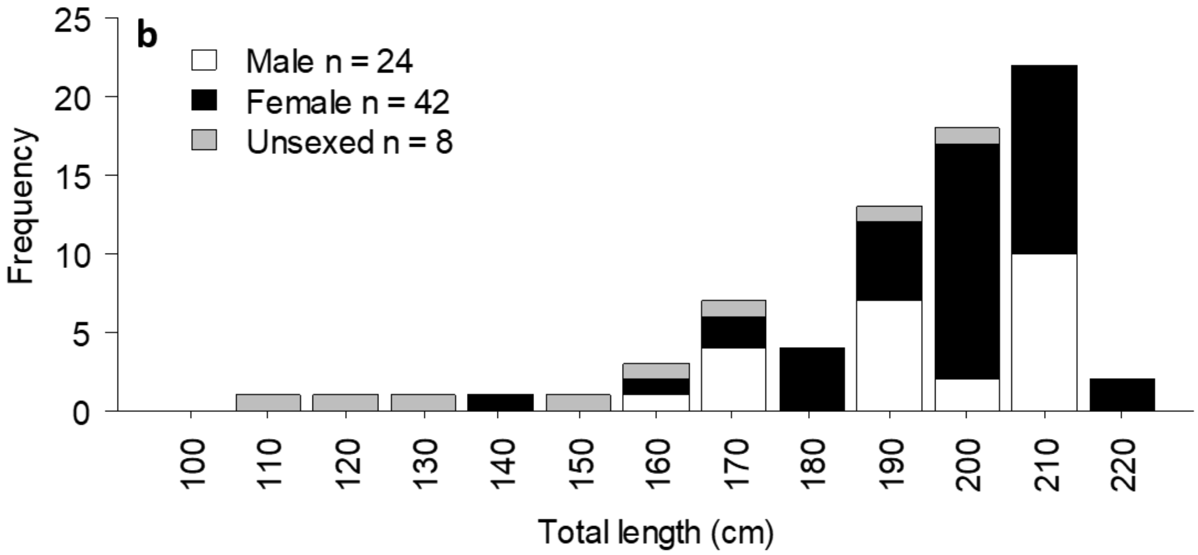

Supplementary Fig. S3. Length-frequency distributions of male (white), female (black) and unknown-sex (grey) bottlenose dolphins sampled in a) south-west and b) Shark Bay study sites. Length bins represent the 10 cm prior to the bin in question, e.g. the '110' bin represents lengths between 100-110cm.

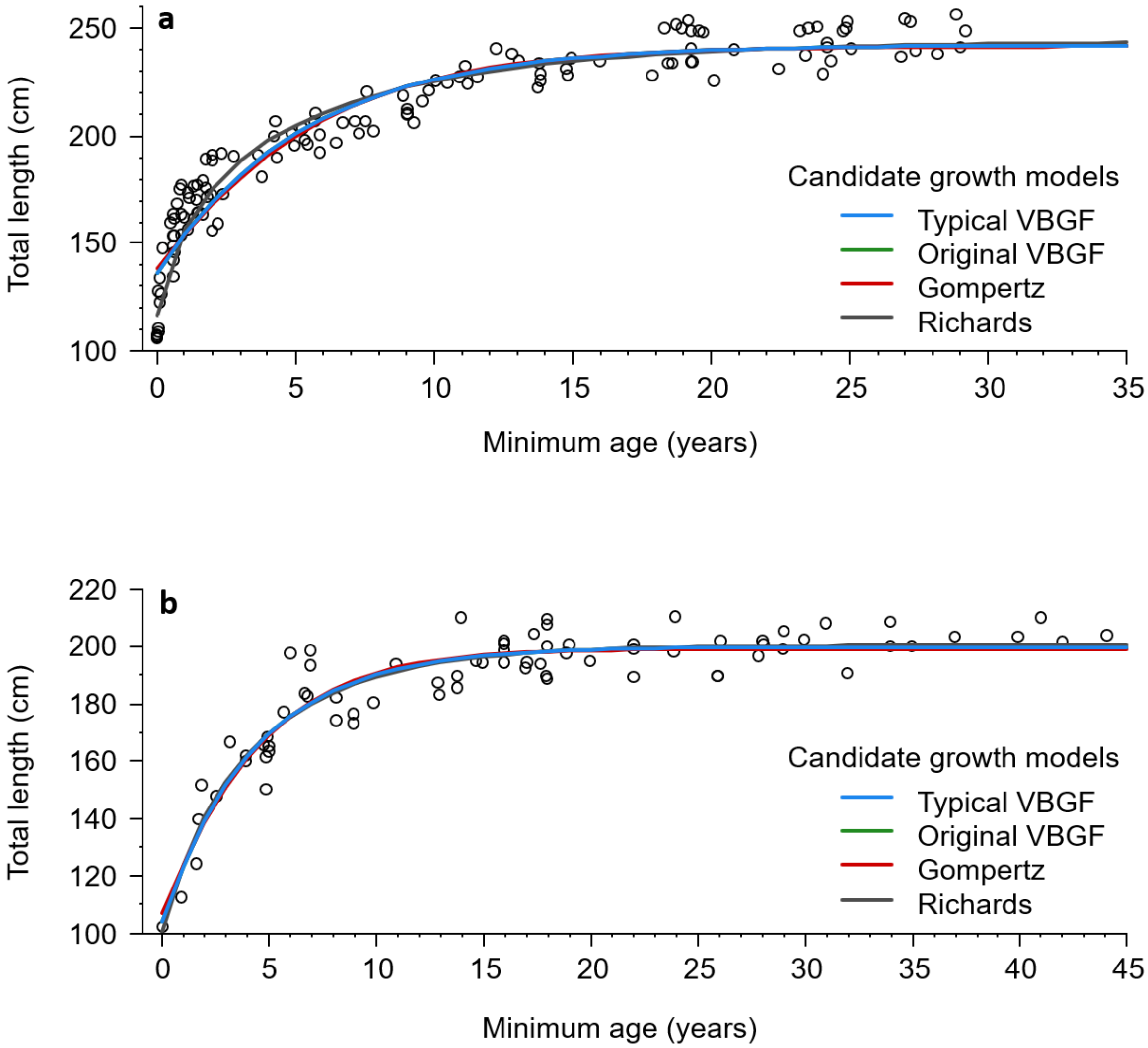

Supplementary Fig. S4. Visual representations of each of the candidate model fits (see legend) relative to the length-at-age data collected in each study site (A, south-west, n = 129; B, Shark Bay, n = 74).

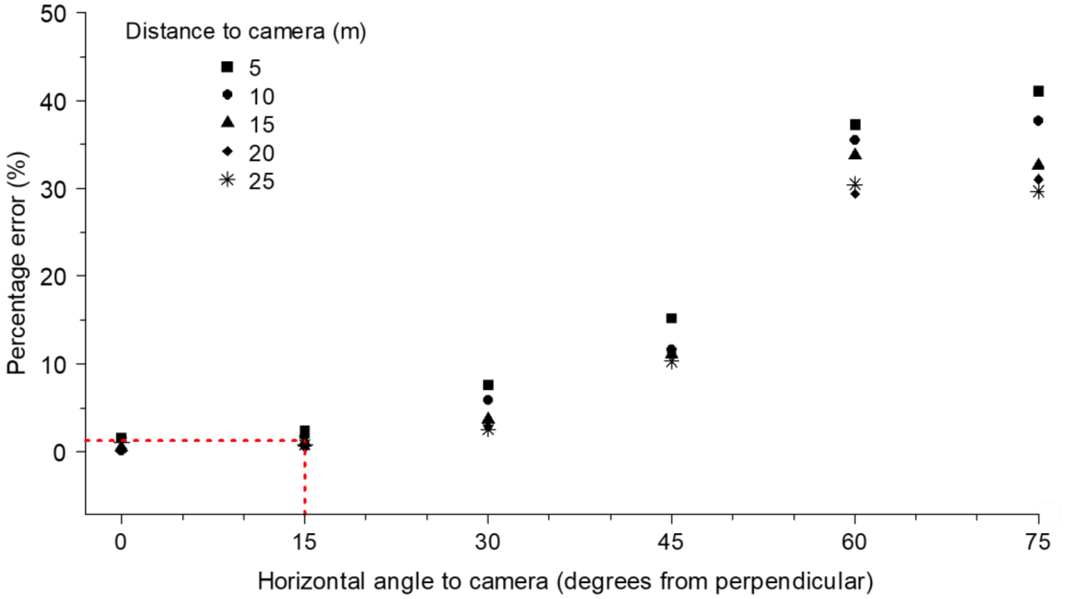

Supplementary Fig. S5. Measurement error results from the three-dimensional dolphin replica experiment. At horizontal angles of 0 - 75°, measurements of a known distance were made in 15° increments over five intervals (5 - 25 m; n = 270). The red dashed lines highlight the mean measurement error value of 1.27% (0.84 cm) obtained at an angle of 15° from perpendicular.

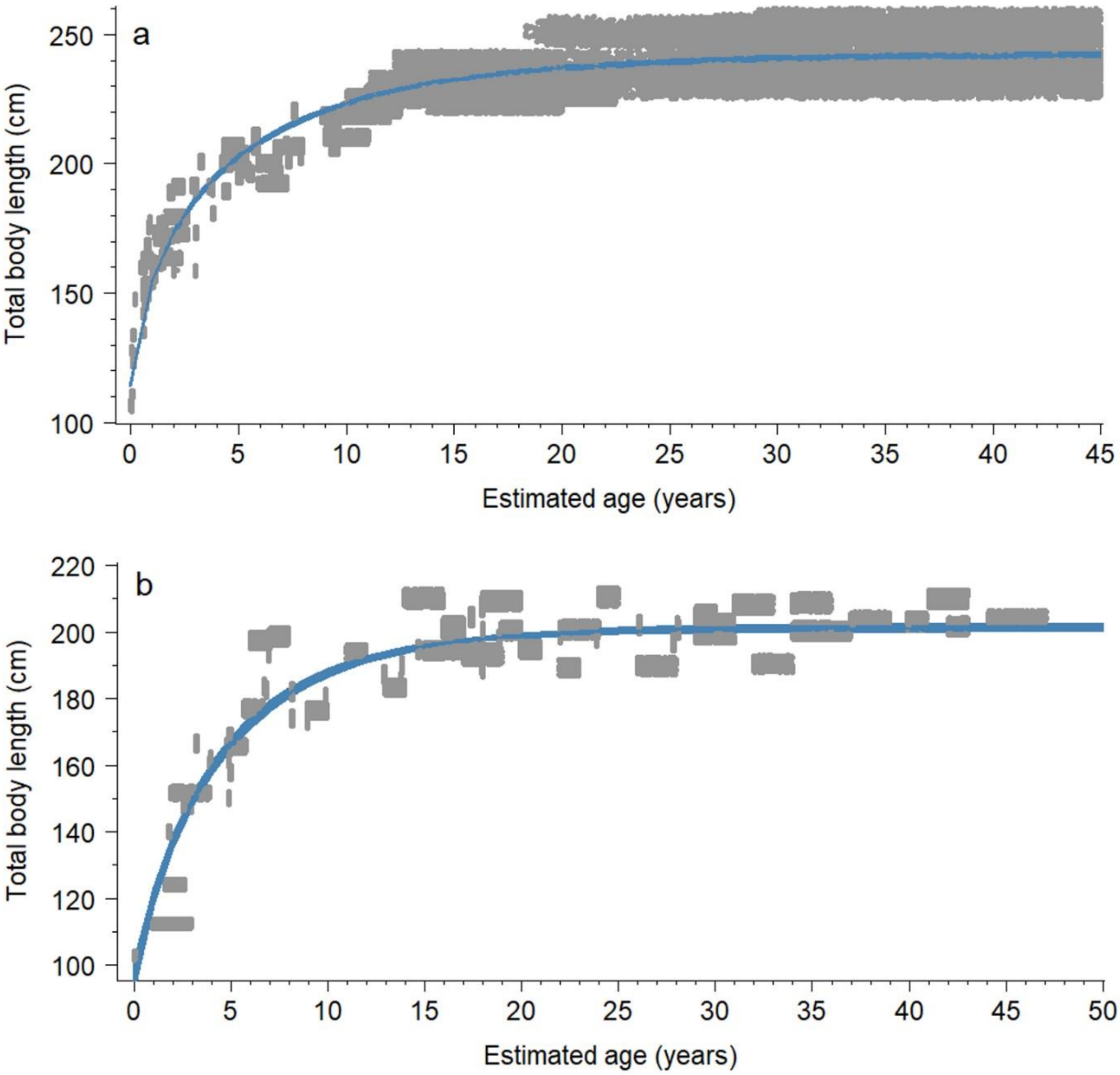

Supplementary Fig. S6. Sensitivity analysis: bootstrapped distributions (grey) characterising the relative measurement and age-estimation error associated with each individual sampled in the south-west (a) and Shark Bay (b) study site. Bootstrapped Richards growth curves are displayed in blue.

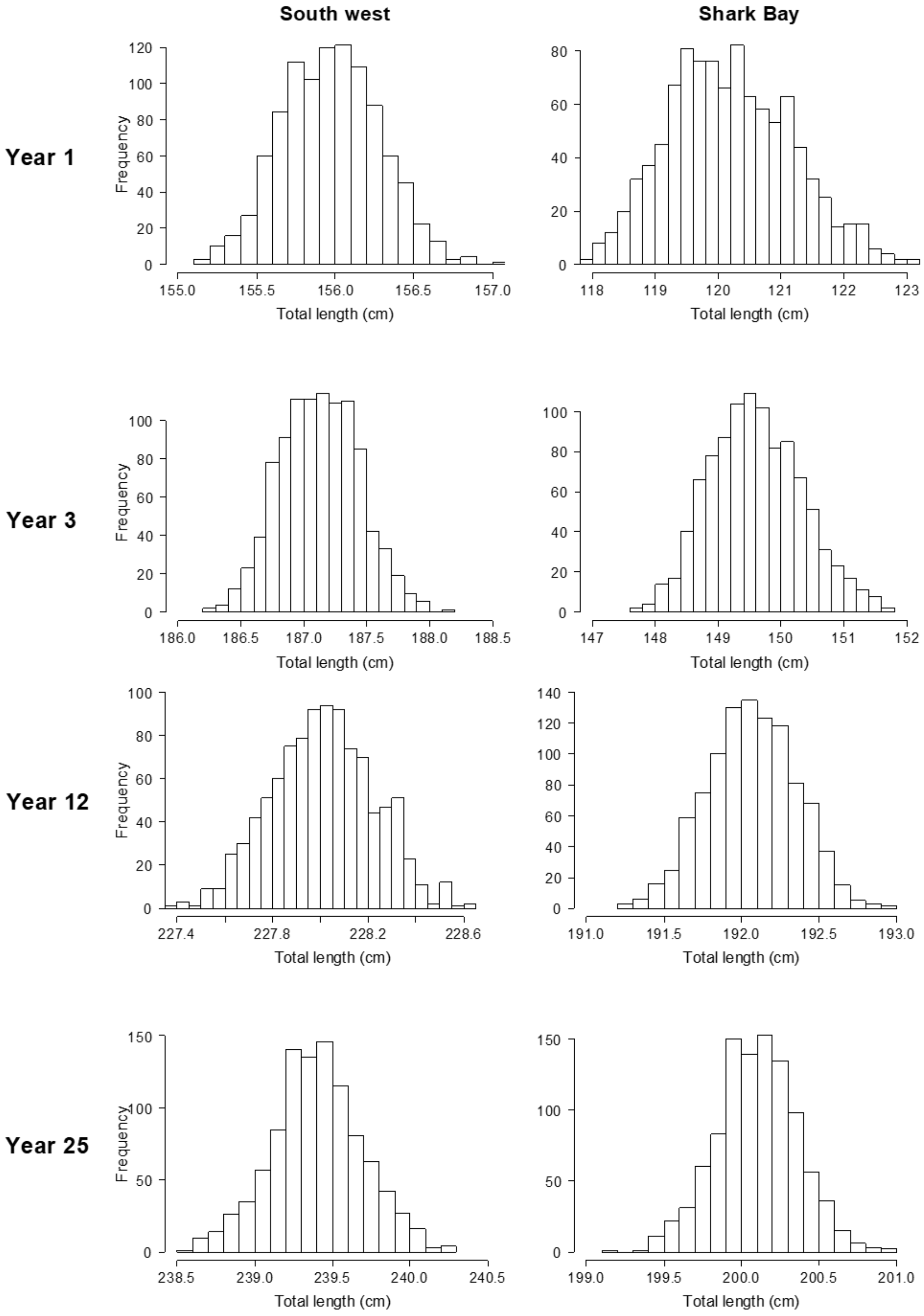

Supplementary Fig. S7. Sensitivity analysis of potential measurement and age-estimation error, with frequency distributions of model parameter values for total length of south-west and Shark Bay dolphins at ages 1, 3, 12, and 25, obtained from 1,000 bootstrap iterations.

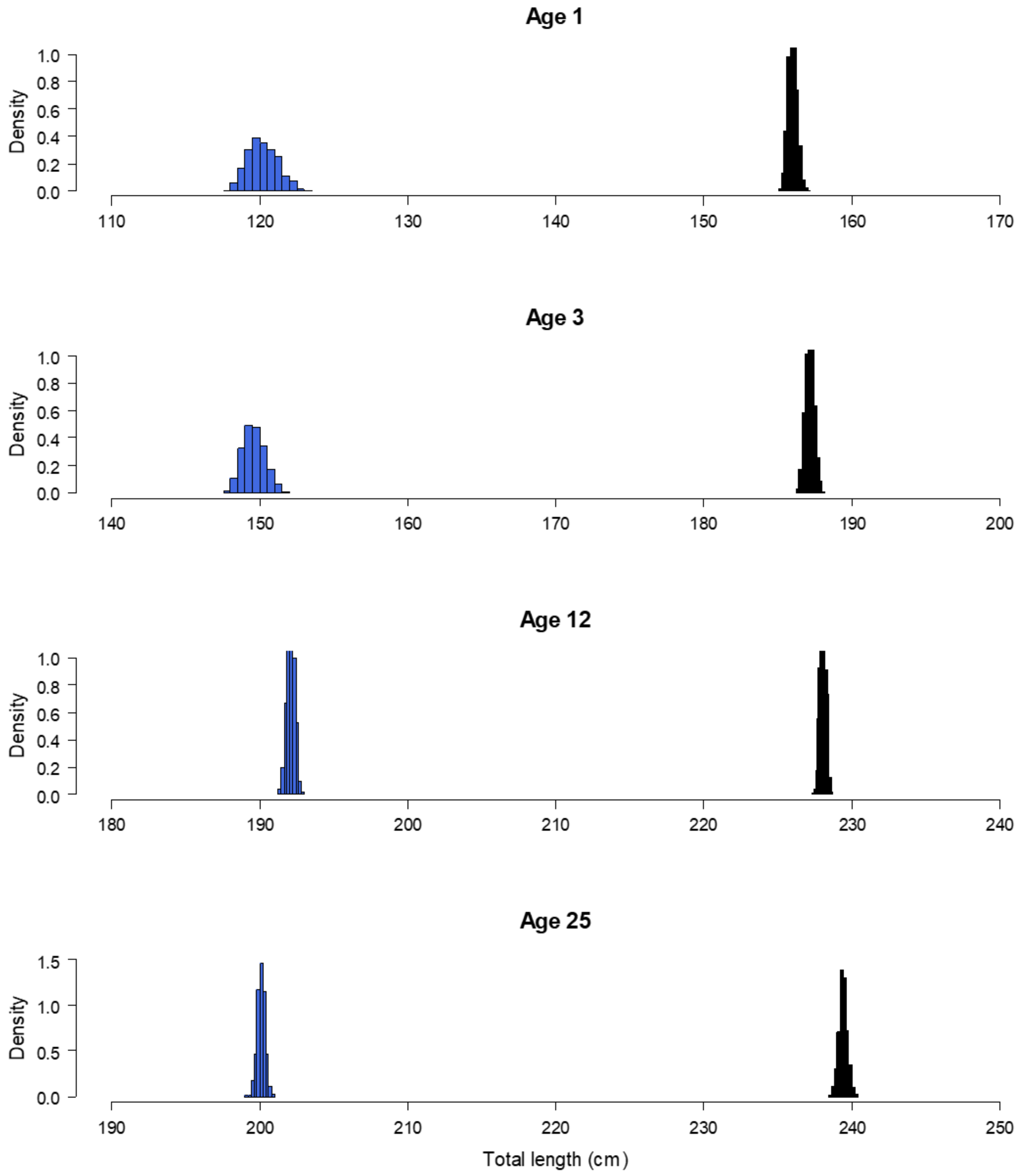

Supplementary Fig. S8. Bootstrapped (1,000 iterations) density distributions of model length-at-age values across the four tested ages: 1, 3, 12, and 25 years. Note significant differences in total length between the south-west (black) and Shark Bay (blue) regions, demonstrated by the lack of overlapping distributions across length.

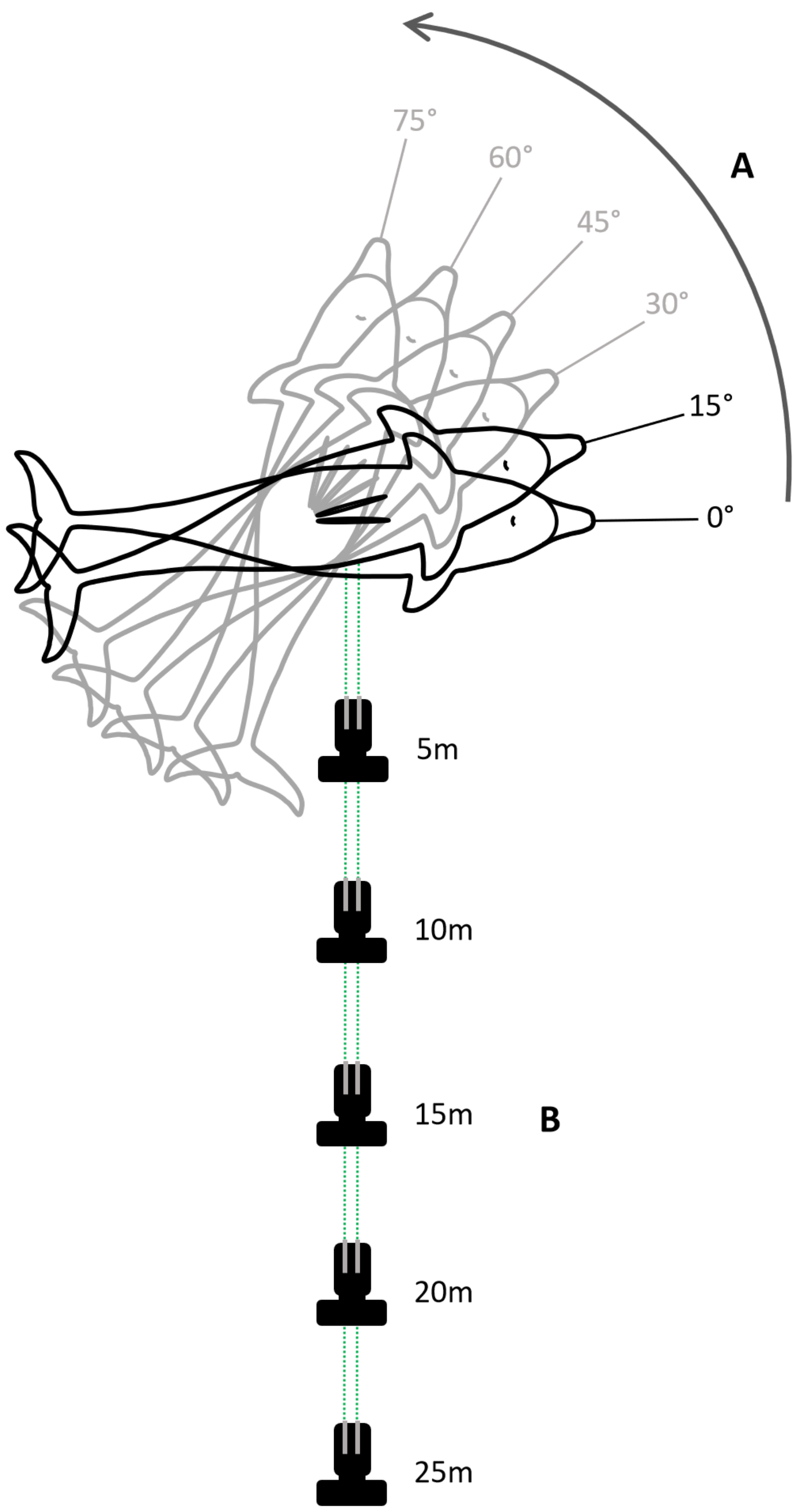

Supplementary Fig. S9. Schematic description of the 3D bottlenose dolphin (*T. aduncus*) replica experiment used to test the influence of horizontal angle and distance on measurement accuracy and precision. The dolphin replica (TL = 210 cm) was rotated in 15° increments (a), with angles at 15° or less from perpendicular (black dolphins) used in image selection. Photographs were taken at each angle in five distance increments between 5 - 25m (b).

LITERATURE CITED

Cheney, B., Wells, R. S., Barton, T. R. & Thompson, P. M. Laser photogrammetry reveals variation in growth and early survival in free-ranging bottlenose dolphins. *Anim Conserv*. **21,** 252-261 (2018).

Karniski, C. *et al.* A comparison of survey and focal follow methods for estimating individual activity budgets of cetaceans. *Mar Mamm Sci*. **31,** 839-852 (2015).
